# Supplementary material for: Non-Conserved Amino Acid Residues Modulate the Thermodynamics of Zn(II) Binding to Classical ββα Zinc Finger Domains
Source: Int J Mol Sci. 2022 Nov 23;23(23):14602. doi: 10.3390/ijms232314602 (PMC9735795; doi:10.3390/ijms232314602)
Supplement: Supplementary file 1 [file ijms-23-14602-s001.zip › ijms-2041423-supplementary.pdf]

Supporting Information

Non-conserved amino acid residues modulate the thermodynamics of Zn(II) binding to classical ββα zinc finger domains

Katarzyna Kluska, Aleksandra Chorążewska, Manuel David Peris-Díaz, Justyna Adamczyk and Artur Krężel*

Department of Chemical Biology, Faculty of Biotechnology, University of Wrocław, Joliot-Curie 14a, 50-383 Wrocław, Poland

\

*To whome correspondence should be addressed

Figures

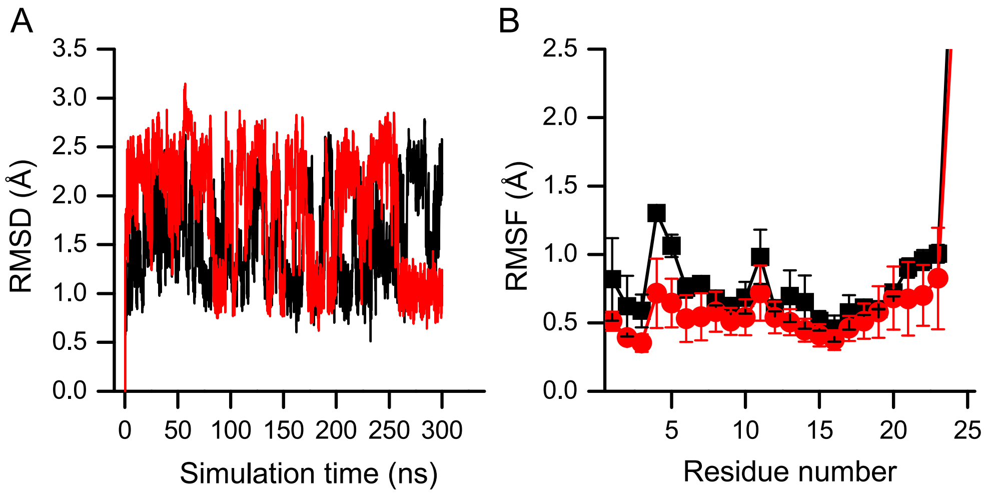

Figure S1. Root mean square deviation (RMSD) plot (A) and root mean square fluctuation (B) as a function of the simulation time assayed and the residue number for the zinc fingers CP1-1991 (black line) and CP1-2015 (red line). The error bar shown in (B) is calculated from three independent production runs of 300 ns.

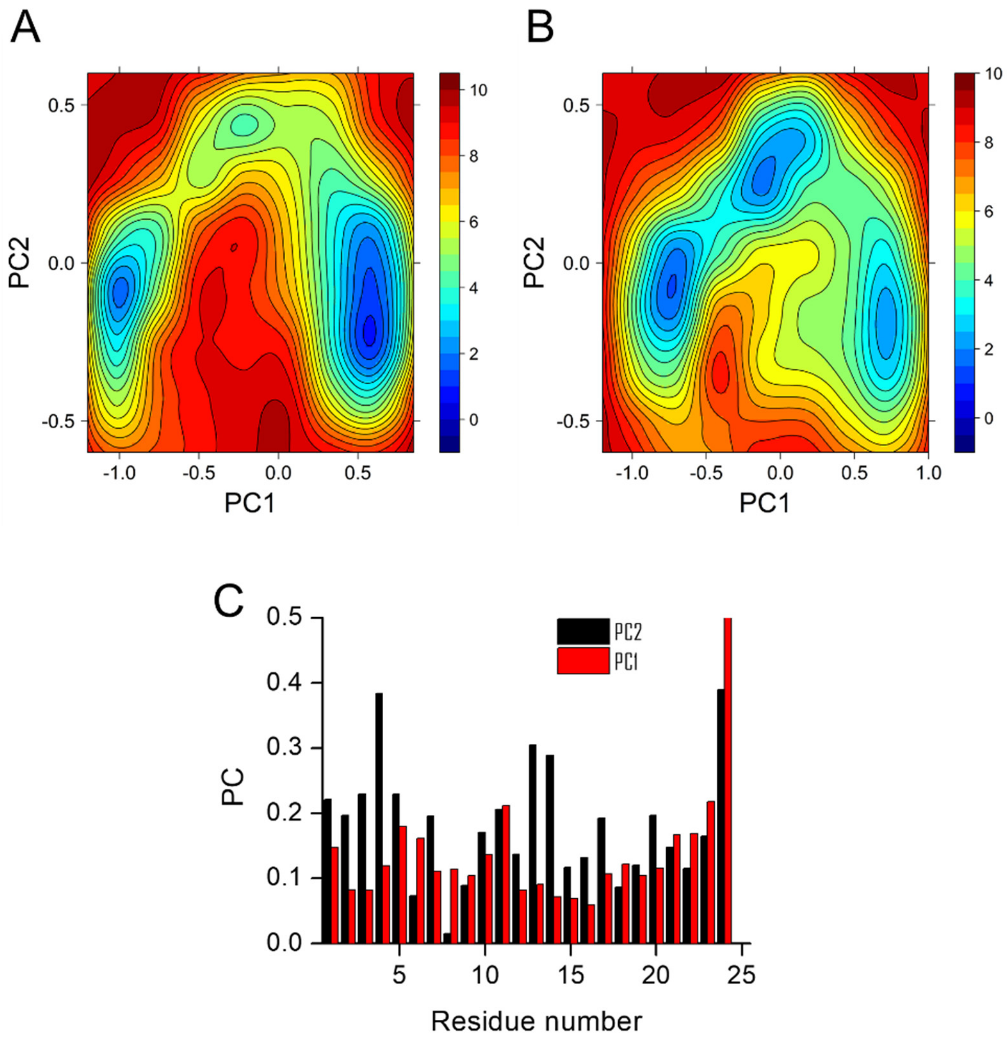

Figure S2. Estimated free energy surface (FES) using as reaction coordinates the principal components 1 and 2 for CP1-2015 (A) and CP1-1991 (B). Principal component (PC) values for PC1 and PC2 for CP1-1991 (C). Principal component (PC) 1 was related to flexibility in the C-terminus whereas the N-terminus and the middle part of the protein contributed the most to PC2. However, no differences were clearly observed between CP1-1991 and CP1-2015.

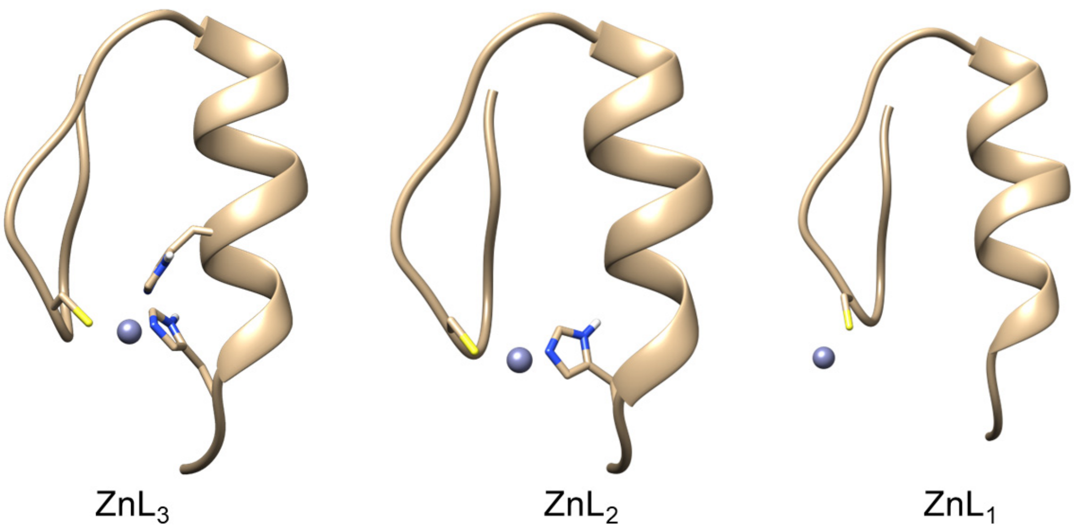

Figure S3. Zn(II) unbinding mechanism obtained from Steered MD simulations. The ZF CP1-1991 was used for representation, but the mechanism can be considered as general taking into consideration the Zn(II)-His swap.

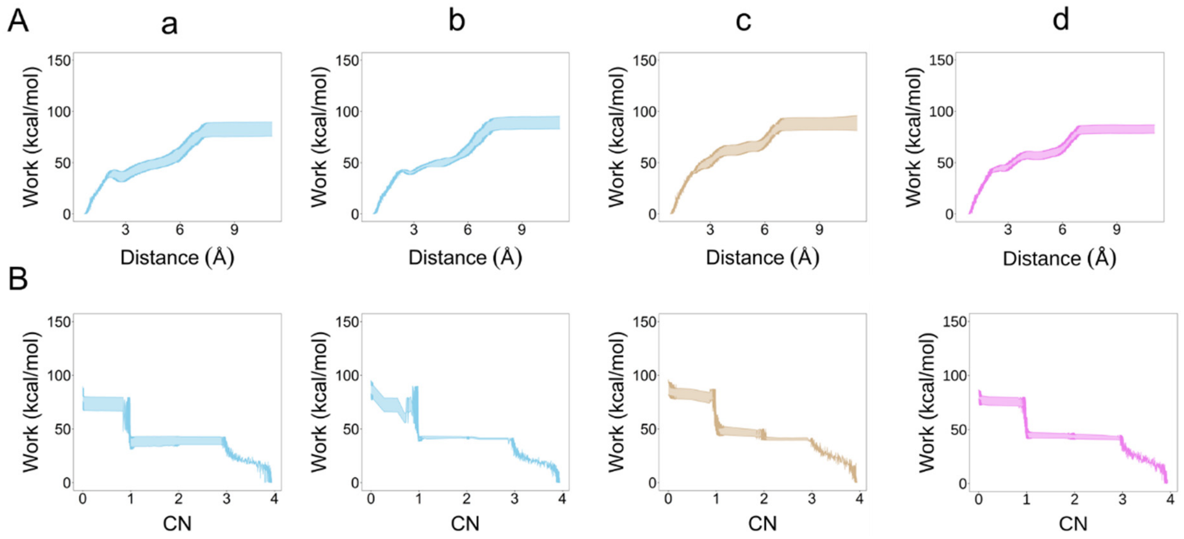

Figure S4. Steered MD simulations for the ZFs CP1-2015 and CP1-1991. (A) Work-extension curve CP1-1991(a), CP1-2015 pathway I (b) and pathway II (c) and for CP1-1991-α (d). (B) Work-extension curve for CP1-1991(a), CP1-2015 pathway I (b) and pathway II (c) and for CP1-1991-α (d) as a function of the contact number (CN).

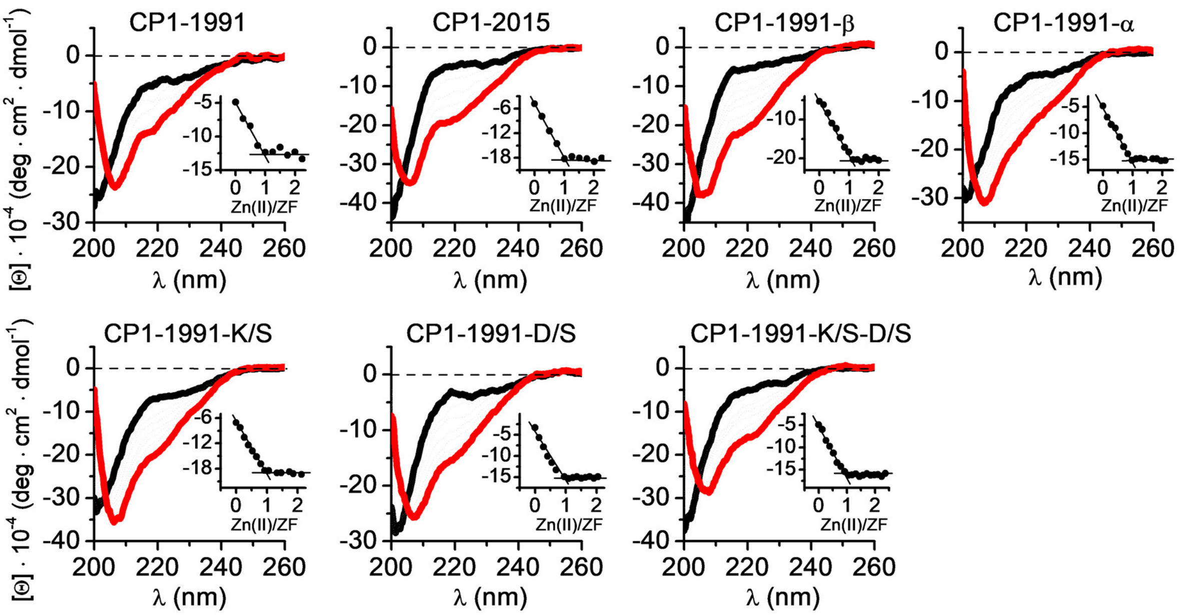

Figure S5. Spectropolarimetric titrations of CP1 ZF peptides with Zn(II). CD spectra of 25 μM ZF peptide in 20 mM Tris-HCl buffer (100 mM NaCl, pH 7.4) in the presence of 0 eq. (red), 0.5 eq. (green) and 1 eq. (blue) of $ZnSO_4$. The insets show the changes in ellipticity at 222 nm over the range of 0–2 Zn(II) eq.

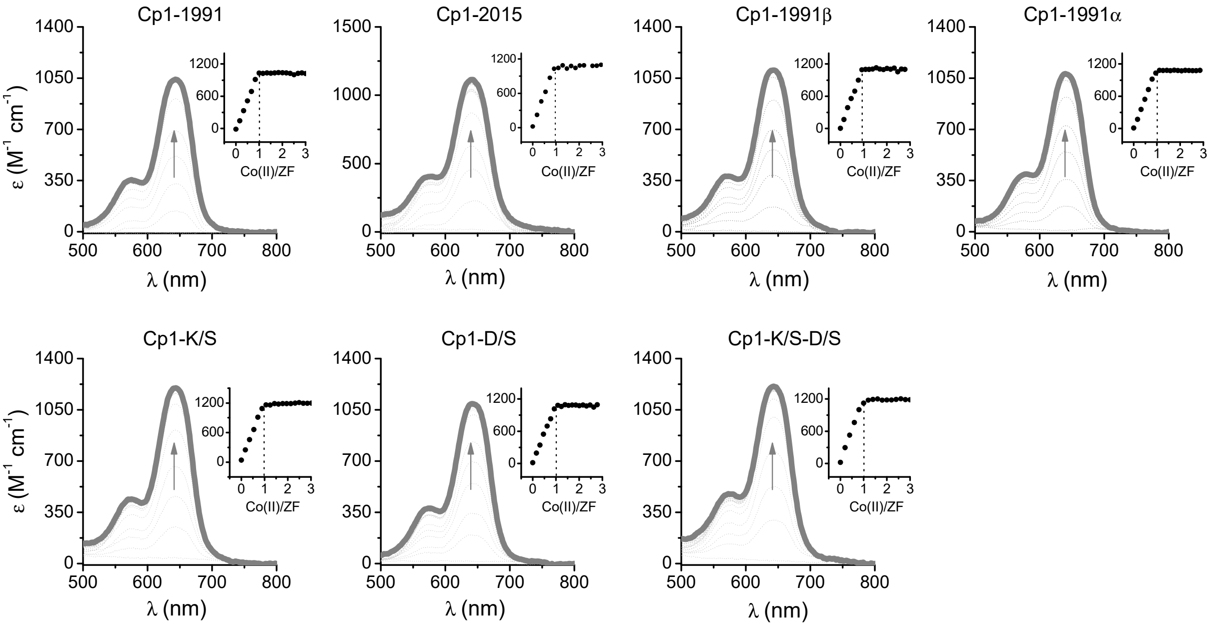

Figure S6. Spectrophotometric titration of CP1 ZF peptides with Co(II). The spectra recorded in the visible range (500–800 nm) of 25 μM CP1 ZF peptide in 50 mM HEPES buffer I = 0.1 M (from NaCl) pH 7.4 in the presence of 0–75 μM Co(II). Absorbance values were converted to molar absorption coefficients based on peptide concentration. Insets represent absorbance increase at maximum wavelength as a function of Co(II)-to-ZF peptide molar ratio.

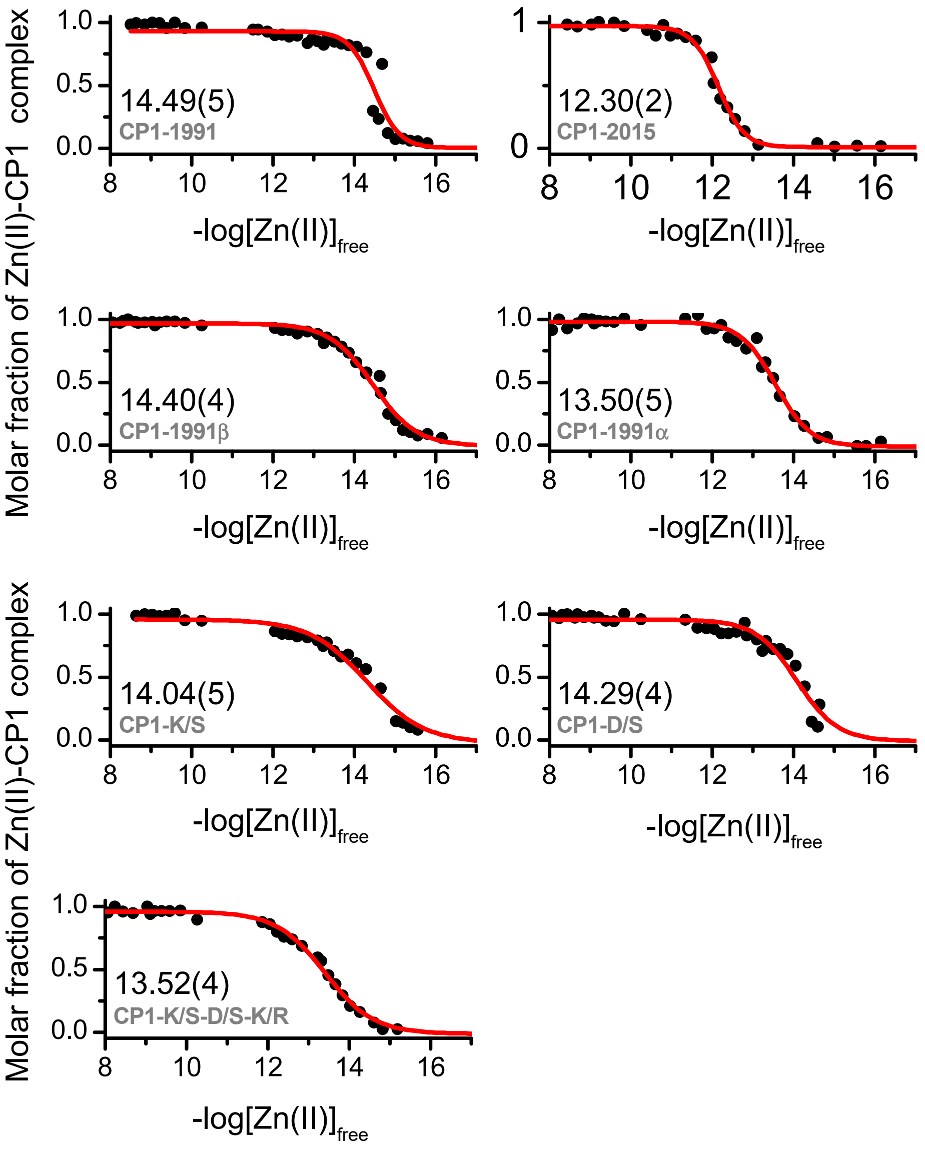

Figure S7. Isotherms of Zn(II) binding to studied CP1 ZF peptides in a set of metal buffers in 20 mM Tris–HCl buffer I = 0.1 M (from NaCl) pH 7.4. The fraction of the ZnZF complex was calculated based on the CD changes in the applied -log[Zn(II)]$_{free}$ range. Red lines represent fits to logarithmic Hill's equation. The values presented in a particular graph are fit results and correspond to -logK_d of the ZnZF complex.

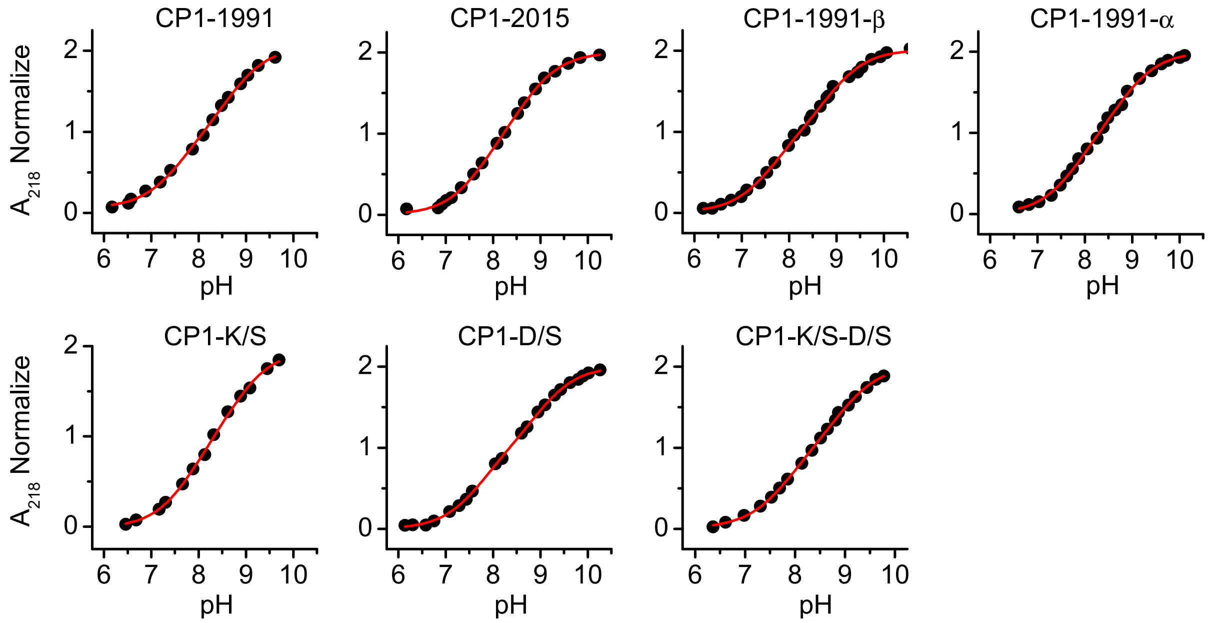

Figure S8. The pH-dependent absorption increase at 218 nm of 30 μM of CP1 ZF peptides, 25˚C, *I* = 0.1 M (from NaCl). The relative best-fitting curves (red) obtained from fitting to two binding event equations (Eq. S3).

Tables

Table S1. Calculated and experimental molecular masses of synthesised peptides that were used in this work. MW_{cal} (calculated) and MW_{exp} (experimental) values refer to averaged, not monoisotopic values obtained through ESI-MS spectra deconvolution.

ZF peptide	MW_{cal} (g/mol)	MW_{exp} (g/mol)
CP1-1991	2906.3	2906.3
CP1-2015	2894.3	2894.6
CP1-1991β	2921.3	2921.1
CP-1991α	2879.2	2879.2
CP1-1991-K/S	2865.2	2865.4
CP1-1991-D/S	2878.2	2878.0
CP1-1991-K/S-D/S	2837.2	2837.0

Table S2. Steered MD simulation results for the Zn(II) dissociation from Zn(II)-loaded ZFs. Numbers 1 to 4 denote the order of residue unbinding in the metal-peptide complex. Frequency occurrence refers to the percentage of times this mechanism was observed for 40 independent runs. Independent simulations that match a particular pathway were grouped, and the rupture force and total work done were average.

ZF	Frequency occurrence (%)	Label pathway	Stepwise Zn-L bond dissociation				Mean rupture force (kcal/mol)	Total work done (kcal/mol)
			1	2	3	4		
CP1-1991	87	a	Cys3	His23	His19	Cys6	16 ± 7	89 ± 7
CP1-2015	35	b	Cys3	His19	His23	Cys6	9 ± 4	82 ± 6
CP1-2015	45	c	Cys3	His23	His19	Cys6	9 ± 4	81 ± 7
CP1-1991a	85	d	Cys3	His23	His19	Cys6	15 ± 7	83 ± 4

Table S3. Spectroscopic properties of Co(II) complexes with altered ZF peptides investigated in this work in 50 mM HEPES, $I = 0.1$ M (from $NaClO_4$), and pH 7.4.

ZF peptide	Complex stoichiometry	Binding residues	Maximum band wavelength (nm)	ε_{max} (M^{-1} cm^{-1})
CP1-1991	ML	CCHH	579	350
CP1-2015	ML	CCHH	579	405
CP1-1991β	ML	CCHH	574	405
CP1-1991α	ML	CCHH	579	400
CP1-1991-K/S	ML	CCHH	576	446
CP1-1991-D/S	ML	CCHH	576	386
CP1-1991-K/S-D/S	ML	CCHH	575	455

Table S4. Acid dissociation constants of cysteine thiol (pK_a^{SH}) groups of zinc finger peptides determined in this work. The abbreviation n.d. denotes not determined.

ZF peptide	pK_{a1}^{SH} N-terminal	pK_{a2}^{SH} C-terminal
CP1-1991	7.54 ± 0.04	9.02 ± 0.04
CP1-2015	7.70 ± 0.03	9.04 ± 0.03
CP1-1991β	7.59 ± 0.03	9.18 ± 0.03
CP1-1991α	7.76 ± 0.03	9.15 ± 0.03
CP1-1991-K/S	7.77 ± 0.03	9.15 ± 0.04
CP1-1991-D/S	7.69 ± 0.02	9.31 ± 0.01
CP1-1991-K/S-D/S	7.77 ± 0.02	9.26 ± 0.02
